# Toward Two-Dimensional Tessellation through Halogen Bonding between Molecules and On-Surface-Synthesized Covalent Multimers

**DOI:** 10.3390/ijms241411291

**Published:** 2023-07-10

**Authors:** David Peyrot, Fabien Silly

**Affiliations:** CEA, CNRS, SPEC, TITANS, Université Paris-Saclay, F-91191 Gif sur Yvette, France; david.peyrot@saint-gobain.com

**Keywords:** molecule, self-assembly, on-surface synthesis, covalent Ullmann coupling, tessalion, tilings

## Abstract

The ability to engineer sophisticated two-dimensional tessellation organic nanoarchitectures based on triangular molecules and on-surface-synthesized covalent multimers is investigated using scanning tunneling microscopy. 1,3,5-Tris(3,5-dibromophenyl)benzene molecules are deposited on high-temperature Au(111) surfaces to trigger Ullmann coupling. The self-assembly into a semi-regular rhombitrihexagonal tiling superstructure not only depends on the synthesis of the required covalent building blocks but also depends on their ratio. The organic tessellation nanoarchitecture is achieved when the molecules are deposited on a Au(111) surface at 145 ${\;}^{{}^{\circ}}C$. This halogen-bonded structure is composed of triangular domains of intact molecules separated by rectangular rows of covalent dimers. The nearly hexagonal vertices are composed of covalent multimers. The experimental observations reveal that the perfect semi-regular rhombitrihexagonal tiling cannot be engineered because it requires, in addition to the dimers and intact molecules, the synthesis of covalent hexagons. This building block is only observed above 165 ${\;}^{{}^{\circ}}C$ and does not coexist with the other required organic buildings blocks.

## 1. Introduction

Atomic-scale engineering of sophisticated two-dimensional (2D) organic nanoarchitectures composed of various building blocks is attracting increasing research interest [1,2,3,4]. This can be achieved by depositing differently shaped molecular building blocks on surfaces and taking advantage of molecular self-assembly through directive and selective intermolecular interactions, such as hydrogen [5,6,7] and halogen bonds [8,9,10,11,12,13,14]. Numerous multicomponent 2D nanoarchitectures have been achieved by taking advantage of hydrogen bonds [15,16,17,18,19,20], but multicomponent 2D nanoarchitectures stabilized by halogen bonds are more scarce.

Ullmann coupling is an appealing reaction for synthesizing new organic building blocks on surfaces by triggering covalent bonds upon the release of molecular halogen atoms [21,22]. Various covalent organic building blocks have thus been synthesized on metal surfaces [23]. It has also been observed that some of them can also self-assemble into 2D structures composed of one organic species [24,25,26].

The engineering of large and well-organized 2D halogen-bonded nanoarchitectures composed of different on-surface-synthesized covalent building blocks appears in comparison to be a lot more challenging, or even purely hypothetical, because the molecular covalent bonding often appears to proceed in a random and an uncontrollable manner. Several bottlenecks have to be overcome to thus engineer this type of 2D multicomponent architecture: the shapes of the different building blocks have to be complementary; the building block ratio has to be adequate; and only the specific halogen atoms have to be released from the molecular skeleton during the Ullmann reaction to trigger the required covalent bonding as well as halogen bonding. The more halogen atoms in the molecule, the more chances increase to trigger irrelevant dehalogenation events. The covalent reaction is, in addition, a non-reversible process in vacuum, thus preventing self-healing, which is essential to reduce the defects in the molecular self-assembly.

**Figure 1:**
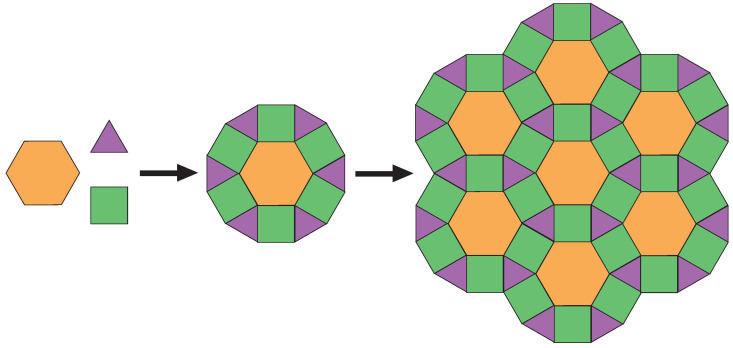
Scheme of the assembly of hexagon, triangle and square patterns (**left**) into a dodecagon (center) and semi-regular rhombitrihexagonal tilings (**right**).

From all the existing 2D organic nanoarchitectures, the semi-regular tilings are some of the most complicated ones to engineer because they can be composed of numerous differently shaped units, which are arranged edge-by-edge. For example, the semi-regular rhombitrihexagonal tiling results from the side-by-side arrangement of triangles, hexagons and squares (Figure 1). Only a few 2D molecular regular tessellations, stabilized by hydrogen bonds, halogen bonds, metal coordination or van der Waals interactions, have already been observed [27,28,29,30,31,32,33,34], resulting from the self-assembly of straight or baton-shaped molecules. Cheng et al. created a 2D manifold complex tessellation by molecular tiles constructed from halogen-bonded and halogen–metal coordinated domains by depositing circular molecules with iodine atoms on Au(111) [34]. This hexagonal superstructure was composed of hexagonal, triangular and rhomboidal units. The authors noticed that the supramolecular tessellation was destroyed after annealing, when temperature-induced intermolecular covalent coupling was triggered by on-surface Ullmann coupling. This experimental observation seems to indicate that the coexistence of molecules with covalent dimers is preventing the formation of 2D tessellation.

**Figure 2:**
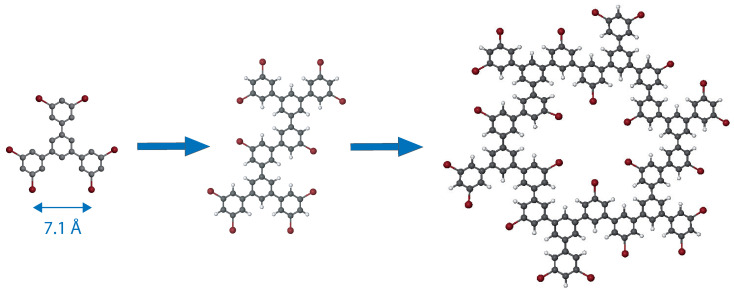
(**Left**) Scheme of the 1,3,5-Tris(3,5-dibromophenyl)benzene (C${}_{24}$H${}_{12}$Br${}_{6}$) molecule, (**center**) covalent dimer, (**right**) covalent hexagon composed of six molecules. Carbon atoms are gray, bromine atoms are red and hydrogen atoms are white.

In this paper, we identify fundamental issues that need to be addressed to engineer large-scale 2D organic semi-regular tessellation through the self-assembly of star-shaped molecules with on-surface-synthesized multimers. 1,3,5-Tris(3,5-dibromophenyl)benzene molecules are deposited on a Au(111) surface at different temperatures to trigger the formation of covalent multimers and molecular self-assembly. Covalent dimers (Figure 2, center) are expected to result from surface-assisted Ullmann coupling between two molecules, whereas covalent hexagons (Figure 2, right) are composed of six covalently linked molecules. The 2D organic nanoarchitectures resulting from the self-assembly of the different building blocks and intermolecular bindings are characterized at the atomic scale using scanning tunneling microscopy.

## 2. Results

### 2.1. Halogen-Bonded Nanoarchitecture of Intact Molecules at Room-Temperature Deposition

**Figure 3:**
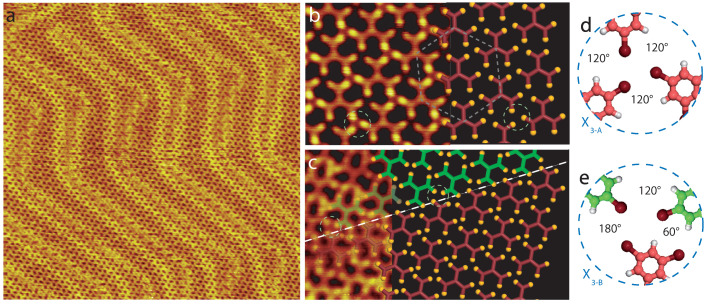
(**a**) STM images of the molecular self-assembly on Au(111)-(22 ×$\sqrt{3}$) at room-temperature deposition, 45 × 45 nm${}^{2}$ (V${}_{s}$ = 1.7 V, I${}_{t}$ = 50 pA). (**b**) Model of the molecular arrangement superimposed onto the STM image, 4 × 4 nm${}^{2}$ (V${}_{s}$ = 1.0 V, I${}_{t}$ = 26 pA). The unit cell is represented by a gray dashed hexagon. (**c**) Model of the molecular arrangement at the domain boundary (highlighted by a dashed line) superimposed onto the STM image, 5 × 3 nm${}^{2}$ (V${}_{s}$ = 2.0 V, I${}_{t}$ = 52 pA). Neighboring domains are represented by red and green molecules. (**d**) X${}_{3-A}$ and (**e**) X${}_{3-B}$ halogen synthons observed inside the molecular arrangement (**b**) and at the domain boundary (**c**), respectively. The angle between the neighboring molecular Br-C axes are mentioned.

Figure 3a shows a large-scale scanning tunneling microscopy (STM) image of 1,3,5-Tris(3,5-dibromophenyl)benzene molecular self-assembly after deposition on a room-temperature Au(111)-(22 × $\sqrt{3}$) surface. The molecules form a large organic layer covering the gold surface. The high-resolution STM image presented in Figure 3b reveals that the molecules self-assemble into a hexagonal arrangement. Only one molecular orientation is observed inside the network. The network unit cell of this structure is a hexagon with a 1.3 nm unit cell constant. This hexagonal unit cell is composed of three molecules. The model of the molecular arrangement is presented in Figure 3b, right. Two bright spots can be observed at the apex of each molecular arm. It has previously been experimentally observed that molecular halogen atoms, such as bromine and iodine, appear brighter than the carbon atoms in STM images [33,35]. The molecules are thus intact when deposited on a room-temperature Au(111) surface. The molecular hexagonal arrangement is stabilized by intermolecular halogen interactions. The molecular bromine atoms are forming X${}_{3}$-synthons (X${}_{3-A}$) with neighboring molecules (Figure 3d). The angle between the Br-C groups of neighboring molecules in X${}_{3-A}$ is 120${\;}^{\circ }$. One of these synthons is highlighted by a dotted circle in the STM image, Figure 3b.

Figure 3c shows a high-resolution STM image of a boundary between neighboring domains. Molecules of neighboring domains are rotated by an angle of 180${\;}^{\circ }$. The model of the molecular arrangement at the domain boundary is presented in Figure 3c, right. Differently oriented molecular schemes (red and green) have been superimposed onto the STM image in Figure 3c as a guide for the eyes. The domain boundary is stabilized by the formation of a second intermolecular X${}_{3}$-synthon (X${}_{3-B}$) between molecules of neighboring domains (dashed circle in Figure 3c). The scheme of this synthon is represented in Figure 3e. The angles between the Br-C groups of neighboring molecules in X${}_{3-B}$ are now 60${\;}^{\circ }$, 120${\;}^{\circ }$ and 180${\;}^{\circ }$.

Bui et al. previously showed that the strength of the halogen bond depends on the C-X⋯X-C bonding angle between neighboring molecules [36]. For an angle of 180${}^{\circ }$, the halogen bond strength is similar to those of van der Waals interactions. It is called a Type-I halogen bond. In comparison, the strength of the halogen bond is stronger and is similar to that of a hydrogen bond, when the C-X⋯X-C angle is close to 90 ± 30${}^{\circ }$. This is called a Type-II halogen bond. The STM image in Figure 3b reveals that the molecular X${}_{3-A}$ synthon is thus only composed of Type-II halogen bonds, as the angle between the three molecular Br-C is 120${}^{\circ }$. In contrast, three angles (60${}^{\circ }$, 120${}^{\circ }$ and 180${}^{\circ }$) are observed between the three neighboring molecular Br-C in X${}_{3-B}$. Therefore, this synthon is composed of one Type-I and two Type-II halogen bonds.

### 2.2. Formation of a 2D Halogen-Bonded Superstructure Composed of Monomers and Covalent Multimers after Deposition Onto a 145 ${\;}^{{}^{\circ}}C$ Surface

**Figure 4:**
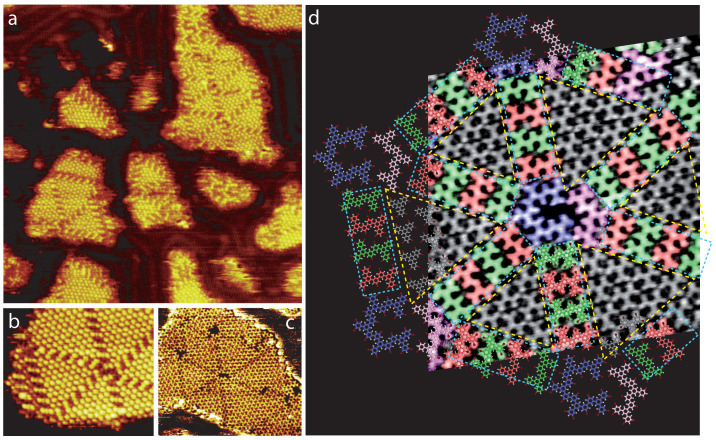
STM images of the molecular self-assembly on Au(111)-(22 ×$\sqrt{3}$) after deposition onto a 145 ${\;}^{\circ }C$ surface: (**a**) 60 × 60 nm${}^{2}$, V${}_{s}$ = 1.0 V, I${}_{t}$ = 15 pA; (**b**) 20 × 20 nm${}^{2}$, V${}_{s}$ = 1.0 V, I${}_{t}$ = 15 pA; (**c**) 30 × 30 nm${}^{2}$, V${}_{s}$ = 1.4 V, I${}_{t}$ = 145 pA; (**d**) 15 × 15 nm${}^{2}$, V${}_{s}$ = 1.4 V, I${}_{t}$ = 145 pA. In (**d**), molecular dimers have been colored in red, green and blue, yellow. Molecular trimers have been colored in dark green, and pentamers in pink. Dotted yellow lines highlight the border of halogen-bonded domains, whereas dotted light-blue lines highlight the side of the two multimers in the center of the image.

Figure 4 shows the Au(111) surface after the deposition of 1,3,5-Tris(3,5-dibromophenyl) benzene molecules on a surface held at 145${\;}^{\circ }C$. The large-scale STM image presented in Figure 4a reveals that molecules now form islands on the surface. In contrast with the self-assembled layer observed at room temperature (Figure 3a), large-scale nearly hexagonal superstructures are visible inside the organic islands. These superstructures are composed of side-by-side triangular domains connected to the same vertex. These superstructures are especially visible in Figure 4a,b, where the contrast in the STM images is inverted. In comparison, the hexagonal superstructures are less noticeable in the standard STM image, Figure 4c.

The STM images reveal that the organic superstructure is nearly hexagonal and its internal arrangement is similar to semi-regular rhombitrihexagonal tilings (Figure 1). The superstructure (Figure 4d) consists of triangular halogen-bonded domains, composed of intact molecules. The molecules (gray color) adopt the arrangement previously observed at room temperature in the self-assembled organic layer, Figure 3b. There is therefore only one molecular orientation inside each triangular domain, but molecules of neighboring triangular domains are rotated by 180${}^{\circ }$. The halogen-bonded triangular domains are separated by rectangular rows composed of covalent dimers (green and red color in Figure 4d) arranged side-by-side. Neighboring triangular domains and dimer rows are pointing toward a nearly hexagonal vertex. The high-resolution STM image in Figure 4d shows that the superstructure vertex is not a hexagon composed of six covalently linked molecules (Figure 2, bottom) but is instead composed of an arch-shaped covalent trimer (pink color) linked to an arch-shaped covalent pentamer (purple color) through halogen bonds. The model of the organic semi-regular rhombitrihexagonal tilings is superimposed onto the STM image in Figure 4d.

**Figure 5:**
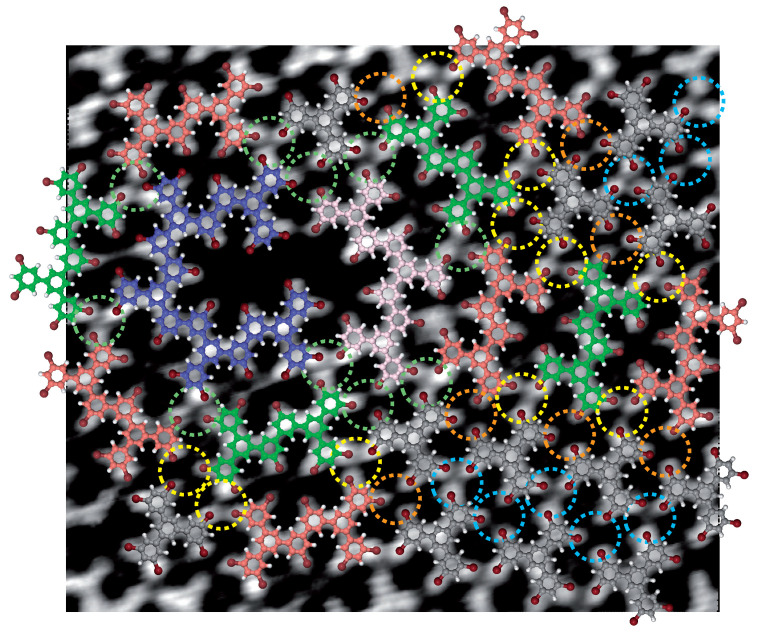
Model of the 2D hybrid superstructure. Schemes of covalent dimers with carbon atoms in orange and green and schemes of covalent trimers with carbon atoms in pink have been superimposed onto an STM image (10 × 8 nm${}^{2}$). Dashed circles highlight the formation of X${}_{3-A}$ synthons.

A close-up investigation of the halogen bonding in the superstructure arrangement is presented in Figure 5. Schemes of molecular covalent dimers (green and red), a covalent trimer (pink), a covalent pentamer (blue) and intact molecules (gray) have been superimposed onto a high-resolution STM image, as a guide for the eyes. The model reveals that the organic superstructure is stabilized by halogen X${}_{3-A}$ synthons only (Figure 3d). As a guide for the eyes, the X${}_{3-A}$ synthons are highlighted by differently colored dashed circles in Figure 5. X${}_{3-A}$ synthons are observed between three intact molecules in the triangular domain (dashed blue circles), as well as between two intact molecules and a covalent dimer (dashed orange circles) and also between one intact molecule and two covalent dimers (dashed yellow circles). The nearly hexagonal-superstructure vertex, located at the intersection between the dimer rows, is composed of two covalent arches. One of the two arches results from the covalent binding of five molecules, whereas the second one results from the covalent binding of three molecules. The arched-shaped covalent multimers of the vertex are also connected to neighboring covalent dimers, a covalent multimer and intact molecules through X${}_{3-A}$ synthons (dashed green circles).

In contrast with the organic layer at room temperature, where X${}_{3-B}$ synthons have been observed at domain boundaries (Figure 3c), only X${}_{3-A}$ synthons are observed in the organic semi-regular rhombitrihexagonal tilings (Figure 5).

**Figure 6:**
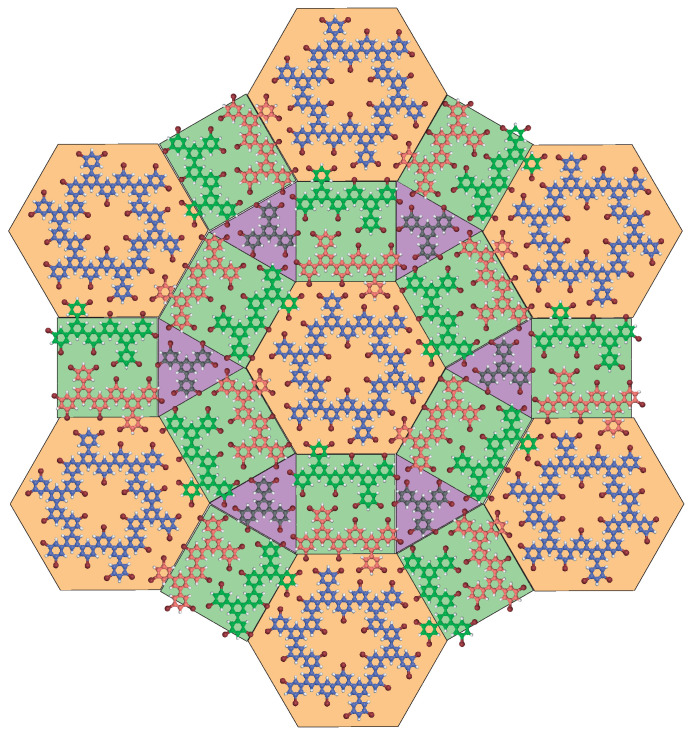
Model of the perfect 2D hybrid superstructure. Carbon atoms of intact molecules are in gray, those of molecular covalent dimers are in orange and green and those of molecular hexagons are in blue. Superimposed purple triangles highlight the domains of intact molecules, whereas yellow hexagons highlight the position of molecular covalent hexagons and green squares the position of covalent dimers.

The model of a perfect semi-regular rhombitrihexagonal tiling based on 1,3,5-Tris(3,5-dibromophenyl)benzene molecules and covalent multimers is presented in Figure 6. To preserve the high symmetry of the superstucture (Figure 1), the hexagonal building blocks in Figure 6 are composed of six-molecule hexagonal multimers (blue), the square building blocks are composed of two covalent dimers (green and red) and the triangular building blocks are composed of single intact molecules. This superstructure is stabilized by X${}_{3-A}$ synthons as experimentally observed in Figure 5.

**Figure 7:**
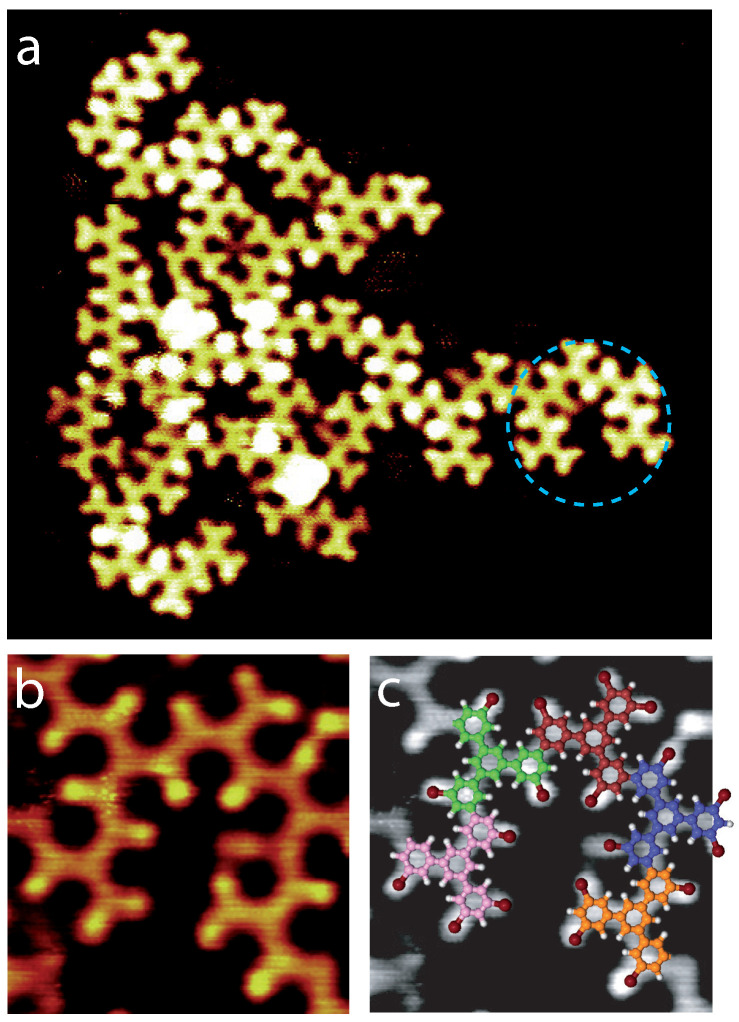
STM images of the molecular self-assembly on Au(111)-(22 ×$\sqrt{3}$) after deposition onto a 165${\;}^{\circ }C$ surface, (**a**) 15 × 14 nm${}^{2}$, V${}_{s}$ = 1.3 V, I${}_{t}$ = 55 pA. (**b**,**c**) Covalent arch arrangements composed of five molecules with superimposed model (pink, green, brown, blue and orange molecules): (**b**) STM image, 3 × 3 nm${}^{2}$, V${}_{s}$ = 1.0 V, I${}_{t}$ = 85 pA, with a superimposed molecular model (**c**).

The perfect semi-regular rhombitrihexagonal tiling based on 1,3,5-Tris(3,5-dibromophenyl) benzene molecules and covalent multimers is presented in Figure 6. This superstructure requires not only the formation of hexagonal covalent multimers, dimers and the presence of intact molecules but also requires a specific ratio between these building blocks. The perfect rhombitrihexagonal tiling is achieved with a building block ratio of one covalent hexagonal vertex for six covalent dimers and two intact molecules, Figure 6. In comparison, the building block ratio in the superstructure obtained after depositing the molecules on a 145${\;}^{\circ }C$ surface is one covalent nearly hexagonal vertex for eight covalent dimers and sixteen intact molecules in Figure 4d. There are therefore too many intact molecules in comparison with the on-surface-synthesized covalent species to achieve the perfect semi-regular rhombitrihexagonal tilings. A higher surface temperature is thus required to trigger the formation of more covalent species, but this is hardly achievable as an increase of only 20${\;}^{\circ }C$ leads to the disappearance of the intact molecules, Figure 7, and thus without triggering the on-surface synthesis of the covalent hexagonal vertices.

A comparison between the ideal model in Figure 6 and the experimental observation in Figure 4 reveals that the dimer rows are perfectly aligned with the row at the opposite side of the hexagonal vertex in the model, whereas there is a shift in the experimental observation. As the vertex is comprised of a covalent pentamer and a covalent trimer in Figure 4, the vertex shape is not perfectly hexagonal, which induces a shift in the alignment of the opposite dimer rows.

In addition, the superstructure model shows that a perfect semi-regular rhombitrihexagonal tiling contains a building block ratio of one covalent hexagon for six covalent dimers and two intact molecules, Figure 6. In contrast, the experimental structure observed in Figure 4 has a building block ratio of one vertex (one pentamer and one trimer) for eight dimers and sixteen intact molecules. Additionally, in the experimental structure, the dimer rows appear as rectangles, whereas they are squares in the model.

### 2.3. Disappearance of 2D Halogen-Bonded Arrangements after Deposition Onto a 165${\;}^{\circ }C$ Surface

The deposition of molecules onto a higher-temperature surface is expected to trigger the formation of covalent hexagons. Figure 7 shows an STM image of the molecular arrangement after deposition onto a Au(111) surface at 165${\;}^{\circ }C$. The hexagonal superstructure, as well as the halogen-bonded arrangements are not visible anymore. The STM image shows that the molecules are now instead forming domains composed of incomplete covalent hexagons (dashed circle in Figure 7a). These incomplete hexagons are mainly composed of five molecules forming an arch, as shown in the high-resolution STM image in Figure 7b and its model in Figure 7c.

### 2.4. Formation of Covalent Hexagons after Deposition Onto a 175${\;}^{\circ }C$ Surface

**Figure 8:**
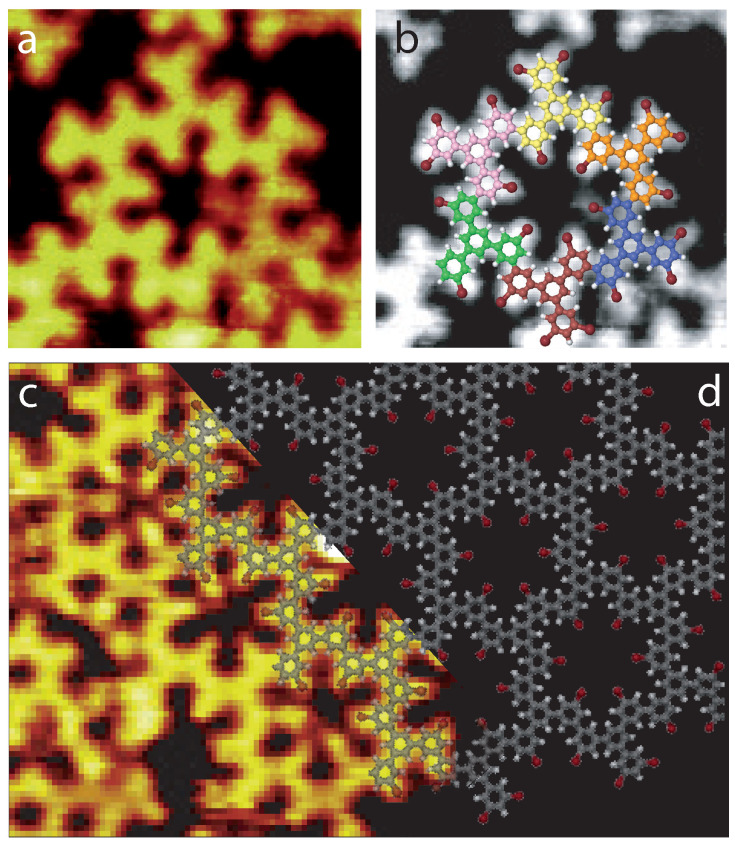
STM images of the molecular arrangement after deposition onto a 175 ${\;}^{\circ }C$ Au(111)-(22 ×$\sqrt{3}$) surface. (**a**) STM image, 4 × 4 nm${}^{2}$, V${}_{s}$ = 1.0 V, I${}_{t}$ = 80 pA, and (**b**) molecular model superimposed onto the STM image. (**c**) STM image, 7 × 6 nm${}^{2}$, V${}_{s}$ = 1.0 V, I${}_{t}$ = 80 pA, and (**d**) molecular model superimposed onto the STM image.

The 1,3,5-Tris(3,5-dibromophenyl)benzene molecules are then deposited onto a 175${\;}^{\circ }C$ Au(111) surface. The STM image in Figure 8a shows that complete covalent hexagons are now created on the surface. Each hexagon is composed of six covalently linked molecules (pink, yellow, orange, blue, brown and green molecular schemes in Figure 8b). Two-dimensional porous covalent-hexagon nanoarchitectures are also experimentally observed on the surface (Figure 8c, left). The model of this structure is superimposed onto the STM image in Figure 8d. The distance separating two hexagon centers is ∼19 Å.

## 3. Discussion

The influence of the Au(111) surface temperature on the self-assembly of 1,3,5-Tris(3,5-dibromophenyl)benzene molecules and on-surface synthesis of covalent multimers has been investigated using STM. The molecules remain intact when deposited on a room-temperature surface and self-assemble into a halogen-bonded hexagonal network, Figure 3b. This organic arrangement is stabilized by X${}_{3-A}$ halogen synthons, Figure 3d. At the domain boundary, molecular domains are rotated by 180 ± 30${}^{\circ }$ (Figure 3c) and a second X${}_{3-B}$ halogen synthon is observed, Figure 3e. The geometry of the X${}_{3-A}$ and X${}_{3-B}$ synthons is slightly different. The Br-C angle between neighboring molecules is 120${}^{\circ }$ in the X${}_{3-A}$ synthon, whereas 60${}^{\circ }$, 120${}^{\circ }$ and 180${}^{\circ }$ angles are observed in the X${}_{3-B}$ synthon. The X${}_{3-A}$ synthon inside the molecular network is thus only composed of a Type-II halogen bond, whereas the X${}_{3-B}$ synthon at the domain boundary is composed of one Type-I and two Type-II halogen bonds [36].

Deposition of molecules onto a 145${\;}^{\circ }C$ Au(111) surface leads to the formation of a nearly 2D hexagonal tiling superstructure, Figure 4. This superstructure is composed of triangular halogen-bonded domains, composed of intact molecules, separated by rows of covalent dimers. The triangular domains and dimer rows meet at nearly hexagonal vertices, composed of covalent multimers. These covalent multimers adopt an arch shape and are usually composed of three-to-five covalently linked molecules. This organic superstructure is stabilized by X${}_{3-A}$ synthons only, Figure 5. For the superstructure to be perfectly symmetrical (as presented in the model in Figure 6), perfect six-molecule covalent hexagons are required at the superstructure vertices, where dimer rows intersect. However, this covalent hexagonal building block is not observed when molecules are deposited on a 145${\;}^{\circ }C$ Au(111) surface.

Covalent molecular chains with an internal five-covalent-molecule-arch geometry (Figure 7) are observed when molecules are deposited on a 165${\;}^{\circ }C$ Au(111) surface; however, no six-covalent-molecule hexagon is created. Additionally, halogen-bonded arrangements of intact molecules are not observed anymore on the surface. This means that building blocks of intact molecules and six-molecule covalent hexagons do not coexist after molecular depositing onto a 165${\;}^{\circ }C$ Au(111) surface.

Figure 8 reveals that the formation of perfect six-covalent-molecule hexagons (Figure 8) is only triggered after depositing the molecules onto a 175${\;}^{\circ }C$ Au(111) surface, a temperature where intact molecules are not subsisting on the surface anymore. It should also be noted that at 175${\;}^{\circ }C$, covalent hexagons are not present as isolated building blocks but exist as covalent nanoarchitectures, Figure 8c.

These observations of the temperature-triggered molecular self-assembly and temperature-triggered intermolecular covalent binding reveal that the “perfect” hexagonal hybrid superstructure presented in Figure 6 cannot be engineered on the Au(111) surface. The STM images show that the temperature required to trigger the formation of a covalent hexagon (175${\;}^{\circ }C$) is so high that intact molecules are not subsisting on the surface anymore. At this temperature, the molecules are fully or at least partially dehalogenated and form covalent bonds with neighboring molecules through Ullmann coupling. No halogen-bonded structures of single 1,3,5-Tris(3,5-dibromophenyl)benzene molecules are then present on the surface. These STM observations show that there is no temperature window, which allows for the coexistence of molecular covalent hexagons and non-covalently linked molecules (intact molecules). This is the reason why the “perfect” hexagonal hybrid tiling superstructure is not observed, Figure 6. The STM images however reveal that despite the fact that the molecular covalent hexagons and non-covalently linked molecules do not coexist after deposition onto a 145${\;}^{\circ }C$ surface, the self-healing process of molecular self-assembly still allows for the formation of molecular tessellation. At 145${\;}^{\circ }C$, the on-surface-synthesized covalent multimers adopt an arch shape and self-assemble into “nearly” hexagonal building blocks, which effectively replace the covalent hexagon of the perfect tiling superstructure. The coexistence of these building blocks with intact molecules and molecular dimers allows for the formation of molecular tessellation, through halogen-bonded self-assembly (Figure 5). The resulting tessellation is thus composed of nearly equilateral triangular domains, resulting from the self-assembly of intact molecules, separated by rows of covalent dimers, Figure 4. The triangular domain corners and dimer rows meet at the nearly hexagonal vertices, which are themselves composed of covalent arched multimers.

It therefore appears that there is no temperature window that satisfies the synthesis and presence of the required organic building blocks to generate the perfect semi-regular rhombitrihexagonal tilings. Nevertheless, organic tiling superstructures, having different building block ratios and where covalent hexagons are replaced by covalent arched multimers, can surprisingly still be created through halogen-bonded self-assembly at 145${\;}^{\circ }C$, Figure 4.

## 4. Materials and Methods

**Sample preparation**: Experiments are performed in an ultrahigh vacuum (UHV) chamber at a pressure of 10${}^{-8}$ Pa. The Au(111) surface is sputtered with Ar${}^{+}$ ions and then annealed in UHV at 600${\;}^{\circ }C$ for 1 hour. The 1,3,5-Tris(3,5-dibromophenyl)benzene molecules (TCI Europe), Figure 2, top-left, are evaporated at 230${\;}^{\circ }C$ and deposited on the gold surface.

**STM imaging:** Cut Pt/Ir tips are used to obtain constant-current STM images at room temperature with a bias voltage applied to the sample. STM images are processed and analyzed using the homemade FabViewer application [37].

**Materials**: The chemical structure of the 1,3,5-Tris(3,5-dibromophenyl)benzene molecule is presented in Figure 2, left. The molecular skeleton of this 3-fold symmetric molecule consists of a central benzene ring connected to three peripheral 3,5-dibromophenyl groups. The bromine atoms of neighboring molecular arms are separated by 7.1 Å.

## 5. Conclusions

To summarize, scanning tunneling microscopy was used to investigate the formation of two-dimensional tessellation organic nanoarchitectures based on 1,3,5-Tris(3,5-dibromophenyl) benzene triangular molecules and on-surface-synthesized covalent multimers on Au(111). Although perfect semi-regular rhombitrihexagonal tilings cannot be achieved, supramolecular halogen-bonded tiling can be engineered at specific temperatures, where intact molecules and covalent multimers are coexisting. These observations open new opportunities for engineering sophisticated high-symmetry 2D nanoarchitectures on metal surfaces. Future work will focus on elucidating the structure-dependent electronic properties of these organic nanoarchitectures using Angle-Resolved Photoemission Spectroscopy.

## Abbreviations

The following abbreviations are used in this manuscript:

STM Scanning tunneling microscopy
